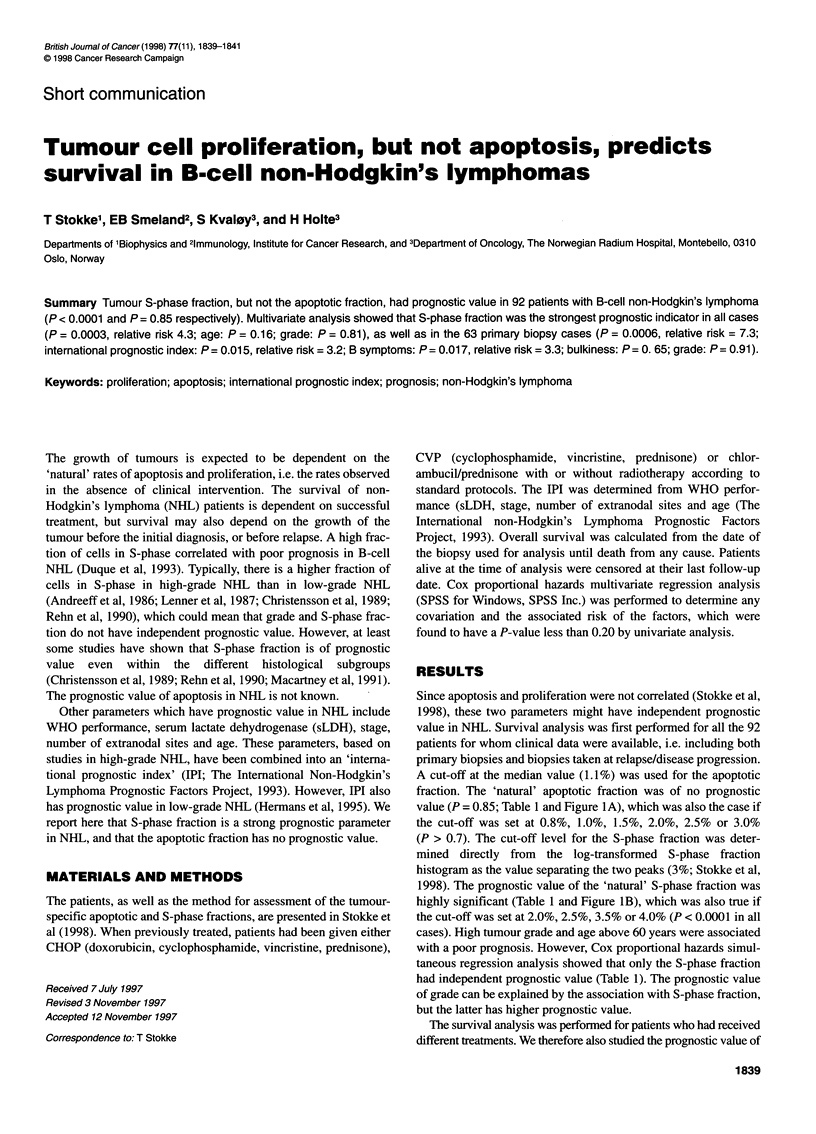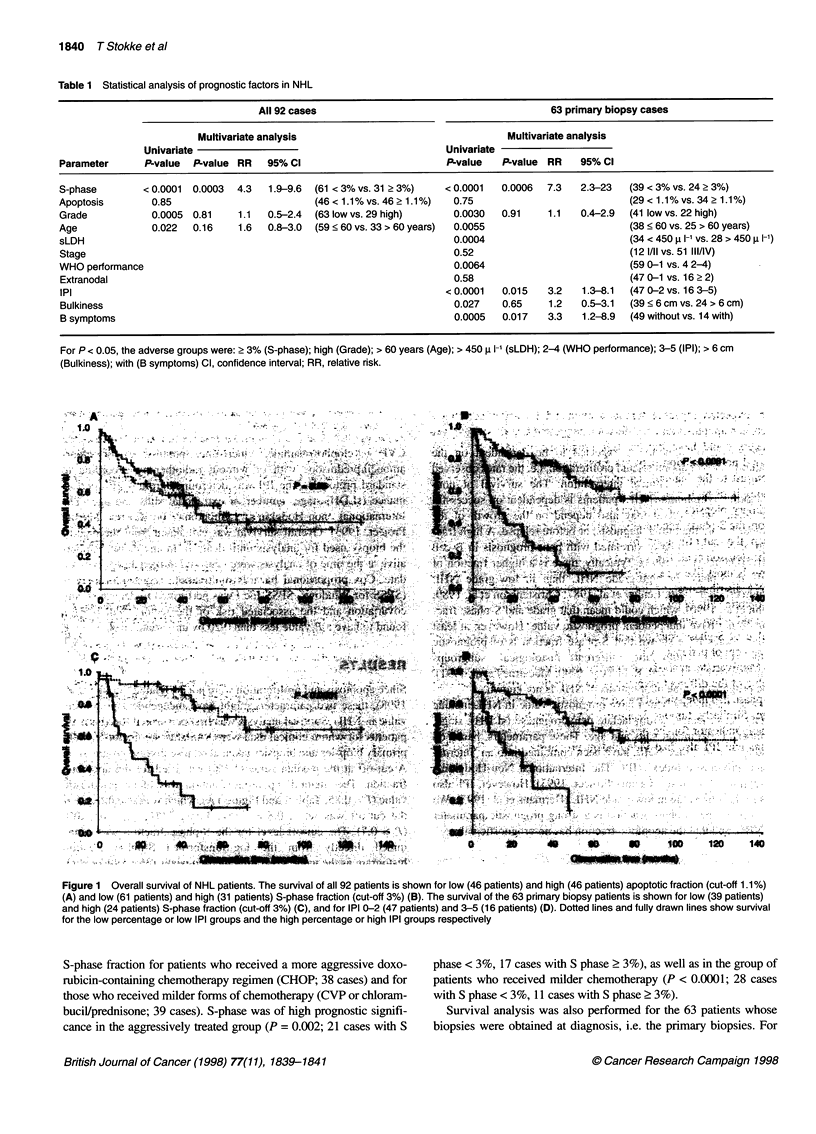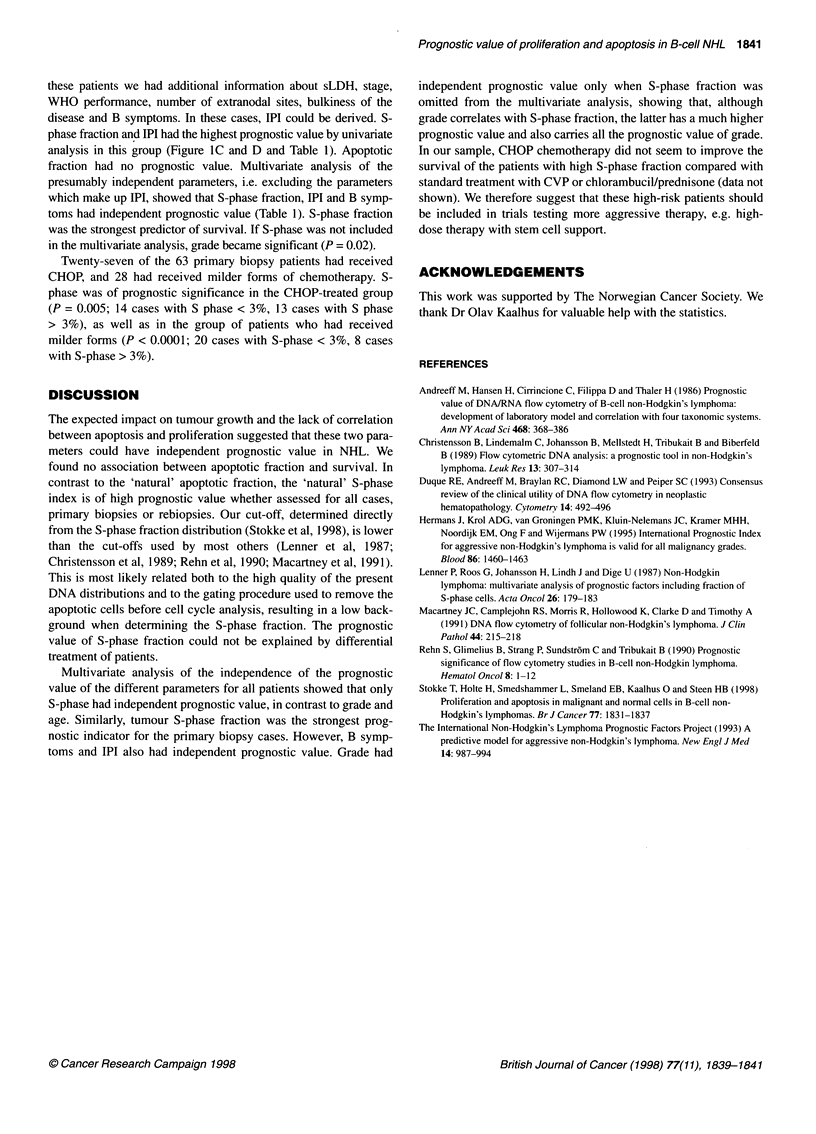# Tumour cell proliferation, but not apoptosis, predicts survival in B-cell non-Hodgkin's lymphomas.

**DOI:** 10.1038/bjc.1998.305

**Published:** 1998-06

**Authors:** T. Stokke, E. B. Smeland, S. KvalÃ¸y, H. Holte

**Affiliations:** Department of Biophysics, The Norwegian Radium Hospital, Montebello.

## Abstract

Tumour S-phase fraction, but not the apoptotic fraction, had prognostic value in 92 patients with B-cell non-Hodgkin's lymphoma (P < 0.0001 and P = 0.85 respectively). Multivariate analysis showed that S-phase fraction was the strongest prognostic indicator in all cases (P = 0.0003, relative risk 4.3; age: P = 0.16; grade: P = 0.81), as well as in the 63 primary biopsy cases (P = 0.0006, relative risk = 7.3; international prognostic index: P = 0.015, relative risk = 3.2; B symptoms: P = 0.017, relative risk = 3.3; bulkiness: P = 0.65; grade: P = 0.91).


British Journal of Cancer (1998) 77(11), 1839–1841
© 1998 Cancer Research Campaign

Short communication

### Tumour cell proliferation, but not apoptosis, predicts survival in B-cell non-Hodgkin's lymphomas

**T Stokke[1], EB Smeland[2], S Kvaløy[3], and H Holte[3]**

Departments of [1]Biophysics and [2]Immunology, Institute for Cancer Research, and [3]Department of Oncology, The Norwegian Radium Hospital, Montebello, 0310 Oslo, Norway

**Summary** Tumour S-phase fraction, but not the apoptotic fraction, had prognostic value in 92 patients with B-cell non-Hodgkin's lymphoma ($P < 0.0001$ and $P = 0.85$ respectively). Multivariate analysis showed that S-phase fraction was the strongest prognostic indicator in all cases ($P = 0.0003$, relative risk 4.3; age: $P = 0.16$; grade: $P = 0.81$), as well as in the 63 primary biopsy cases ($P = 0.0006$, relative risk = 7.3; international prognostic index: $P = 0.015$, relative risk = 3.2; B symptoms: $P = 0.017$, relative risk = 3.3; bulkiness: $P = 0.65$; grade: $P = 0.91$).

**Keywords:** proliferation; apoptosis; international prognostic index; prognosis; non-Hodgkin's lymphoma

The growth of tumours is expected to be dependent on the 'natural' rates of apoptosis and proliferation, i.e. the rates observed in the absence of clinical intervention. The survival of non-Hodgkin's lymphoma (NHL) patients is dependent on successful treatment, but survival may also depend on the growth of the tumour before the initial diagnosis, or before relapse. A high fraction of cells in S-phase correlated with poor prognosis in B-cell NHL (Duque et al, 1993). Typically, there is a higher fraction of cells in S-phase in high-grade NHL than in low-grade NHL (Andreeff et al, 1986; Lenner et al, 1987; Christensson et al, 1989; Rehn et al, 1990), which could mean that grade and S-phase fraction do not have independent prognostic value. However, at least some studies have shown that S-phase fraction is of prognostic value even within the different histological subgroups (Christensson et al, 1989; Rehn et al, 1990; Macartney et al, 1991). The prognostic value of apoptosis in NHL is not known.

Other parameters which have prognostic value in NHL include WHO performance, serum lactate dehydrogenase (sLDH), stage, number of extranodal sites and age. These parameters, based on studies in high-grade NHL, have been combined into an 'international prognostic index' (IPI; The International Non-Hodgkin's Lymphoma Prognostic Factors Project, 1993). However, IPI also has prognostic value in low-grade NHL (Hermans et al, 1995). We report here that S-phase fraction is a strong prognostic parameter in NHL, and that the apoptotic fraction has no prognostic value.

### MATERIALS AND METHODS

The patients, as well as the method for assessment of the tumour-specific apoptotic and S-phase fractions, are presented in Stokke et al (1998). When previously treated, patients had been given either CHOP (doxorubicin, cyclophosphamide, vincristine, prednisone), CVP (cyclophosphamide, vincristine, prednisone) or chlorambucil/prednisone with or without radiotherapy according to standard protocols. The IPI was determined from WHO performance (sLDH, stage, number of extranodal sites and age (The International non-Hodgkin's Lymphoma Prognostic Factors Project, 1993). Overall survival was calculated from the date of the biopsy used for analysis until death from any cause. Patients alive at the time of analysis were censored at their last follow-up date. Cox proportional hazards multivariate regression analysis (SPSS for Windows, SPSS Inc.) was performed to determine any covariation and the associated risk of the factors, which were found to have a $P$-value less than 0.20 by univariate analysis.

*Received 7 July 1997*
*Revised 3 November 1997*
*Accepted 12 November 1997*

*Correspondence to:* T Stokke

### RESULTS

Since apoptosis and proliferation were not correlated (Stokke et al, 1998), these two parameters might have independent prognostic value in NHL. Survival analysis was first performed for all the 92 patients for whom clinical data were available, i.e. including both primary biopsies and biopsies taken at relapse/disease progression. A cut-off at the median value (1.1%) was used for the apoptotic fraction. The 'natural' apoptotic fraction was of no prognostic value ($P = 0.85$; Table 1 and Figure 1A), which was also the case if the cut-off was set at 0.8%, 1.0%, 1.5%, 2.0%, 2.5% or 3.0% ($P > 0.7$). The cut-off level for the S-phase fraction was determined directly from the log-transformed S-phase fraction histogram as the value separating the two peaks (3%; Stokke et al, 1998). The prognostic value of the 'natural' S-phase fraction was highly significant (Table 1 and Figure 1B), which was also true if the cut-off was set at 2.0%, 2.5%, 3.5% or 4.0% ($P < 0.0001$ in all cases). High tumour grade and age above 60 years were associated with a poor prognosis. However, Cox proportional hazards simultaneous regression analysis showed that only the S-phase fraction had independent prognostic value (Table 1). The prognostic value of grade can be explained by the association with S-phase fraction, but the latter has higher prognostic value.

The survival analysis was performed for patients who had received different treatments. We therefore also studied the prognostic value of

**Table 1** Statistical analysis of prognostic factors in NHL

| | All 92 cases | | | | | 63 primary biopsy cases | | | | |
|---|---|---|---|---|---|---|---|---|---|---|
| | | Multivariate analysis | | | | | Multivariate analysis | | | |
| **Parameter** | **Univariate *P*-value** | ***P*-value** | **RR** | **95% CI** | | **Univariate *P*-value** | ***P*-value** | **RR** | **95% CI** | |
| S-phase | < 0.0001 | 0.0003 | 4.3 | 1.9–9.6 | (61 < 3% vs. 31 ≥ 3%) | < 0.0001 | 0.0006 | 7.3 | 2.3–23 | (39 < 3% vs. 24 ≥ 3%) |
| Apoptosis | 0.85 | | | | (46 < 1.1% vs. 46 ≥ 1.1%) | 0.75 | | | | (29 < 1.1% vs. 34 ≥ 1.1%) |
| Grade | 0.0005 | 0.81 | 1.1 | 0.5–2.4 | (63 low vs. 29 high) | 0.0030 | 0.91 | 1.1 | 0.4–2.9 | (41 low vs. 22 high) |
| Age | 0.022 | 0.16 | 1.6 | 0.8–3.0 | (59 ≤ 60 vs. 33 > 60 years) | 0.0055 | | | | (38 ≤ 60 vs. 25 > 60 years) |
| sLDH | | | | | | 0.0004 | | | | (34 < 450 μ $l^{-1}$ vs. 28 > 450 μ $l^{-1}$) |
| Stage | | | | | | 0.52 | | | | (12 I/II vs. 51 III/IV) |
| WHO performance | | | | | | 0.0064 | | | | (59 0–1 vs. 4 2–4) |
| Extranodal | | | | | | 0.58 | | | | (47 0–1 vs. 16 ≥ 2) |
| IPI | | | | | | < 0.0001 | 0.015 | 3.2 | 1.3–8.1 | (47 0–2 vs. 16 3–5) |
| Bulkiness | | | | | | 0.027 | 0.65 | 1.2 | 0.5–3.1 | (39 ≤ 6 cm vs. 24 > 6 cm) |
| B symptoms | | | | | | 0.0005 | 0.017 | 3.3 | 1.2–8.9 | (49 without vs. 14 with) |

For $P < 0.05$, the adverse groups were: ≥ 3% (S-phase); high (Grade); > 60 years (Age); > 450 μ $l^{-1}$ (sLDH); 2–4 (WHO performance); 3–5 (IPI); > 6 cm (Bulkiness); with (B symptoms) CI, confidence interval; RR, relative risk.

**Figure 1** Overall survival of NHL patients. The survival of all 92 patients is shown for low (46 patients) and high (46 patients) apoptotic fraction (cut-off 1.1%) (**A**) and low (61 patients) and high (31 patients) S-phase fraction (cut-off 3%) (**B**). The survival of the 63 primary biopsy patients is shown for low (39 patients) and high (24 patients) S-phase fraction (cut-off 3%) (**C**), and for IPI 0–2 (47 patients) and 3–5 (16 patients) (**D**). Dotted lines and fully drawn lines show survival for the low percentage or low IPI groups and the high percentage or high IPI groups respectively

S-phase fraction for patients who received a more aggressive doxorubicin-containing chemotherapy regimen (CHOP; 38 cases) and for those who received milder forms of chemotherapy (CVP or chlorambucil/prednisone; 39 cases). S-phase was of high prognostic significance in the aggressively treated group ($P = 0.002$; 21 cases with S phase < 3%, 17 cases with S phase ≥ 3%), as well as in the group of patients who received milder chemotherapy ($P < 0.0001$; 28 cases with S phase < 3%, 11 cases with S phase ≥ 3%).

Survival analysis was also performed for the 63 patients whose biopsies were obtained at diagnosis, i.e. the primary biopsies. For

© Cancer Research Campaign 1998

these patients we had additional information about sLDH, stage, WHO performance, number of extranodal sites, bulkiness of the disease and B symptoms. In these cases, IPI could be derived. S-phase fraction and IPI had the highest prognostic value by univariate analysis in this group (Figure 1C and D and Table 1). Apoptotic fraction had no prognostic value. Multivariate analysis of the presumably independent parameters, i.e. excluding the parameters which make up IPI, showed that S-phase fraction, IPI and B symptoms had independent prognostic value (Table 1). S-phase fraction was the strongest predictor of survival. If S-phase was not included in the multivariate analysis, grade became significant ($P = 0.02$).

Twenty-seven of the 63 primary biopsy patients had received CHOP, and 28 had received milder forms of chemotherapy. S-phase was of prognostic significance in the CHOP-treated group ($P = 0.005$; 14 cases with S phase < 3%, 13 cases with S phase > 3%), as well as in the group of patients who had received milder forms ($P < 0.0001$; 20 cases with S-phase < 3%, 8 cases with S-phase > 3%).

### DISCUSSION

The expected impact on tumour growth and the lack of correlation between apoptosis and proliferation suggested that these two parameters could have independent prognostic value in NHL. We found no association between apoptotic fraction and survival. In contrast to the 'natural' apoptotic fraction, the 'natural' S-phase index is of high prognostic value whether assessed for all cases, primary biopsies or rebiopsies. Our cut-off, determined directly from the S-phase fraction distribution (Stokke et al, 1998), is lower than the cut-offs used by most others (Lenner et al, 1987; Christensson et al, 1989; Rehn et al, 1990; Macartney et al, 1991). This is most likely related both to the high quality of the present DNA distributions and to the gating procedure used to remove the apoptotic cells before cell cycle analysis, resulting in a low background when determining the S-phase fraction. The prognostic value of S-phase fraction could not be explained by differential treatment of patients.

Multivariate analysis of the independence of the prognostic value of the different parameters for all patients showed that only S-phase had independent prognostic value, in contrast to grade and age. Similarly, tumour S-phase fraction was the strongest prognostic indicator for the primary biopsy cases. However, B symptoms and IPI also had independent prognostic value. Grade had independent prognostic value only when S-phase fraction was omitted from the multivariate analysis, showing that, although grade correlates with S-phase fraction, the latter has a much higher prognostic value and also carries all the prognostic value of grade. In our sample, CHOP chemotherapy did not seem to improve the survival of the patients with high S-phase fraction compared with standard treatment with CVP or chlorambucil/prednisone (data not shown). We therefore suggest that these high-risk patients should be included in trials testing more aggressive therapy, e.g. high-dose therapy with stem cell support.

### ACKNOWLEDGEMENTS

This work was supported by The Norwegian Cancer Society. We thank Dr Olav Kaalhus for valuable help with the statistics.

### REFERENCES

Andreeff M, Hansen H, Cirrincione C, Filippa D and Thaler H (1986) Prognostic value of DNA/RNA flow cytometry of B-cell non-Hodgkin's lymphoma: development of laboratory model and correlation with four taxonomic systems. *Ann NY Acad Sci* **468**: 368–386

Christensson B, Lindemalm C, Johansson B, Mellstedt H, Tribukait B and Biberfeld B (1989) Flow cytometric DNA analysis: a prognostic tool in non-Hodgkin's lymphoma. *Leuk Res* **13**: 307–314

Duque RE, Andreeff M, Braylan RC, Diamond LW and Peiper SC (1993) Consensus review of the clinical utility of DNA flow cytometry in neoplastic hematopathology. *Cytometry* **14**: 492–496

Hermans J, Krol ADG, van Groningen PMK, Kluin-Nelemans JC, Kramer MHH, Noordijk EM, Ong F and Wijermans PW (1995) International Prognostic Index for aggressive non-Hodgkin's lymphoma is valid for all malignancy grades. *Blood* **86**: 1460–1463

Lenner P, Roos G, Johansson H, Lindh J and Dige U (1987) Non-Hodgkin lymphoma: multivariate analysis of prognostic factors including fraction of S-phase cells. *Acta Oncol* **26**: 179–183

Macartney JC, Camplejohn RS, Morris R, Hollowood K, Clarke D and Timothy A (1991) DNA flow cytometry of follicular non-Hodgkin's lymphoma. *J Clin Pathol* **44**: 215–218

Rehn S, Glimelius B, Strang P, Sundström C and Tribukait B (1990) Prognostic significance of flow cytometry studies in B-cell non-Hodgkin lymphoma. *Hematol Oncol* **8**: 1–12

Stokke T, Holte H, Smedshammer L, Smeland EB, Kaalhus O and Steen HB (1998) Proliferation and apoptosis in malignant and normal cells in B-cell non-Hodgkin's lymphomas. *Br J Cancer* **77**: 1831–1837

The International Non-Hodgkin's Lymphoma Prognostic Factors Project (1993) A predictive model for aggressive non-Hodgkin's lymphoma. *New Engl J Med* **14**: 987–994

© Cancer Research Campaign 1998